# Effects of Angelicae Pubescentis and Loranthi Decotion on repairing knee joint cartilages in rats

**DOI:** 10.1186/s13018-017-0679-8

**Published:** 2017-12-12

**Authors:** Shun Lyu, Bin Ji, Wenwu Gao, Xianqi Chen, Xiaotao Xie, Junjie Zhou

**Affiliations:** Department of orthopedics, Putuo Hospital, Shanghai University of Traditional Chinese Medicine, No. 164 Lanxi Rd, Shanghai, 200062 China

**Keywords:** Angelicae Pubescentis and Loranthi decotion, Duhuo, Chondrocyte, Knee osteoarthritis, Serum

## Abstract

**Background:**

Knee osteoarthritis (KOA) is a common orthopedics disease and its pathological changes at early stage are the damage and loss of articular cartilage. Traditional Chinese medicine (TCM) prescription contains multiple components and has the unique advantages of the diversity of targets.We compared the traditional Chinese medical formulae (Angelicae Pubescentis and Loranthi decotion, APLD, or Duhuo Jisheng) with a western medicine (glucosamine sulfate, GS) to treat the rat arthritis models, and tracked the outcomes.

**Methods:**

Thirty-two Wistar rats (weight 180 ± 10 g, 6-week-old) were randomly divided into four groups (eight for each): group A as normal control group (no surgery and no drug treatment), group B as SIA (surgery-induced arthritis) model control without drug treatment, group C as SIA model + APLD, and group D as SIA model + GS. Anterior cruciate ligament in the knee joint of both hind legs from each rat in groups B, C, and D was shown and cut off to establish the SIA model. After 6 weeks of the surgery, rats in group C or D were treated with APLD or GS, respectively, for 8 weeks. Bone X-ray examination, histological images, and determination of genes of collagen II and aggrecan were performed. At week 14, both knee joint gap and bone structure disappeared in rats of group B, but they were visible in rats of groups A, C, and D.

**Results:**

Histological images revealed that the structure and composition of the knee joint cartilage were significantly degenerated in group B and improved in group C. Genes of collagen II and aggrecan were significantly increased in both group C and D.

**Conclusion:**

APLD or GS gavage treatment for knee osteoarthritis (KOA) rat models was effective on the proliferation of cartilage chondrocytes and the damaged knee joint tissue repairing, and the APLD showed slightly superior in general.

## Background

Knee joint is the largest joint of the body. Knee osteoarthritis (KOA) is a common orthopedics disease [1, 2]; its pathological changes at early stage are the damage and loss of articular cartilage. When the knee articular cartilage is damaged, the ability of articular cartilage self-repair is very limited, because of the particularity of its structure and function [3]. The treatment at the early and middle stage of KOA has been focused on the pain relief. However, there has been a problem on the treatment at the early or middle stage, because it is unable to stop the KOA at the early or middle stages and does not inhibit the attenuation, degeneration, and loss of osteochondral layer of the joint, and therefore, it could not prevent the osteoarthritis developing to the late stage. At the late stage, the knee joint has severe deformation; thus, osteo-surgery, such as joint replacement, is the only choice of treatment [4]. However, the main problem of surgery treatment is surgical trauma and unstable healing [5]. Tissue engineering (TE) launched a new path to solve the problem in the treatment of KOA [1]. TE of cartilage could provide better allograft in terms of shape and geometry to match the peripheral tissue; better biomaterial to promote the aggregation, proliferation, and differentiation of chondrocytes for knee joint regeneration; and better microenvironment to mimic the articular regeneration. TE technique has been used to repair cartilage defects.

Traditional Chinese medicine (TCM) prescription contains multiple components and has the unique advantages of the diversity of targets. Moreover, the application of serum with TCM components has been introduced [6]. After experimental animals are administrated with gavage of TCM for a period, the serum of the animals contains the components of the TCM. Such serum is, then, used to culture cells in vitro. Angelicae Pubescentis and Loranthi decoction (APLD, also called Duhuo Jisheng) has been used to treat osteoarthritis in China for thousand years, showing a better pain-relief effect and slowing down of the articular cartilage degeneration [7–9]. The modern pharmacological studies showed that APLD could protect the subchondral bone structure from collagen-induced arthritis in rat models [10], the increase of the extract concentration of APLD led to the increased activity of cartilage cells, and APLD could inhibit the apoptosis of chondrocytes through the expression of mitochondrial factor caspase-3 and caspase-9 [11–13]. In our previous study of serum containing APLD, we successfully inducted the amniotic cells as seed cells to differentiate into chondrocytes [14].

In this study, gavage with APLD as TCM and glucosamine sulfate as western medicine were used to treat the surgery-induced arthritis (SIA) model in rats; the multiple results were compared and analyzed.

## Method

### Drug formula

The raw materials of APLD (Duhuo Jisheng) were purchased from the Chinese Pharmacy of Shanghai University of Traditional Chinese Medicine (Shanghai, China), and the decoction was made at the Pharmacy Department in Putuo Hospital of Shanghai University of Chinese Traditional Medicine. One prescription of APLD contains 15 types of herbals: Duhuo 9 g, Sangjisheng 6 g, Duzhong 6 g, Huainiuxi 6 g, Xixin 6 g, Qinjiao 6 g, Baifuling 6 g, Cinnamon 6 g, Fangfeng 6 g, Chuanxiong 6 g, Maorenshen 6 g, Gancao 6 g, Danggui 6 g, Baishao 6 g, and Gandihuang 6 g. One prescription of APLD produces 200 ml of decoction. Glucosamine sulfate (GS, purchased from Rottapharm Ltd., China) was used as the western medicine to compare with APLD. GS was prepared as solution of 0.012 g/ml. The 0.9% saline (Shanghai Baite, Ltd., China) was used as control group.

### Rat SIA model establishing and drug treatment

Thirty-two healthy Wistar rats (weight 180 ± 10 g, 6-week-old) purchased from Slack Laboratory Animal Co., Ltd. (manufacturing license number: SCXK (Shanghai) 2013–0016, Shanghai, China) were randomly divided into four groups (eight for each): group A as the normal control group (no surgery and no drug treatment), group B as the SIA model control without drug treatment, group C as the SIA model + APLD, and group D as the SIA model + GS. All animal experiments followed the Institutional Animal Care and Use Committee (IACUC) protocol at the Shanghai University of Traditional Chinese Medicine.

**Fig. 1:**
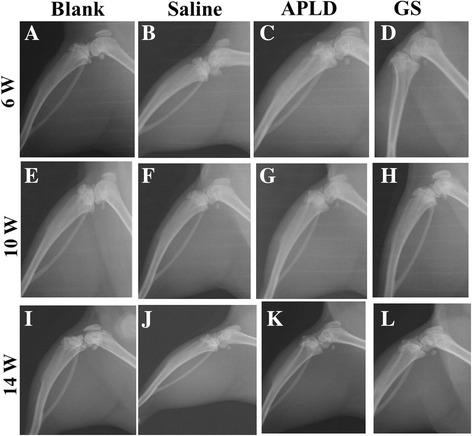
The X-ray images of the right hind leg of rats from each group were taken at 6, 10 and 14 weeks, respectively. **A**, **E**, **I** belong to group A (blank, control group, no surgery and no drug treatment); **B**, **F**, **J** belong to group B (the SIA model control without drug treatment); **C**, **G**, **K** belong to group C (the SIA model + APLD treatment); **D**, **H**, **L** belong to group D (the SIA model + GS treatment)

SIA models were successfully confirmed after 6 weeks of surgery; knee joint X-ray examinations were taken (Fig. 1a–d). Knee joint X-ray examinations were taken at 4 and 8 weeks after drug treatments (that means 10 and 14 weeks after surgery) (Fig. 1e–l) to reveal and compare the different drug effects.

#### Twenty-four rat SIA models

Two percent of pentobarbital sodium of 0.2 ml/100 g rat weight was injected into peritoneal of each rat from groups B, C, and D; fur on bilateral knee joint of hind legs of each rat was shaved, each knee joint was disinfected with 75% alcohol, the incision of 2 cm inside of the patellar was made to expose patellar ligament, patella was located, and fascia tissue was gently torn with a scalpel to straight knee. Then, joint capsule was opened, and anterior cruciate ligament was shown and cut off. Bleeding was stopped, and the patella was reset. The wound was closed and disinfected again. After the surgery, the rats were back to normal life in each group for 6 weeks to establish the SIA models without any drug treatments.

#### Drug treatments

After 6 weeks of SIA model establishment, each rat in group B was filled with 0.9% saline of 4 ml/day; each rat in group C was filled with 4 ml APLD decoction/day, which was calculated based on 0.9 ml of APLD decoction/100 g rat body weight, eliminating lavage loss; and each rat in group D was filled with 4 ml of glucosamine sulfate solution/day (the concentration of glucosamine sulfate solution was 120 mg/ml, to get 1.2 ml/100 g rat body weight). Except lavage loss, each of the rats in each group was filled with two times a day and 2 ml of each time till week 14.

### Collection of knee joint cartilage and serum specimen at week 10 and 14

At 4 and 8 weeks of drug treatments (that means at week 10 and 14), three rats from each group were killed with the fast spinal dislocation method. The blood was aspirated from the abdomen arteries of each rat, kept at room temperature for at least 4 h, and then centrifuged with 3000 and 12,000 RPM sequentially under 4 °C. The supernatant (that is serum) was carefully removed without touching bottom clot. The serum was filtered and then stored in a sterilized container at − 20 °C ready for use. All left knee articular cartilages were collected for qPCR analysis of aggrecan and collagen II mRNA testing, and all right knee articular cartilages were fixed in 4% paraformaldehyde for histologic examination.

### Histological and immonuhistochemical imaging

Rat right knee joints were fixed by 4% paraformaldehyde for 24 h, decalcified with 0.5 M EDTA (Boston Bioproducts, USA) for 2 weeks, and dehydrated with gradient ethanol. Then, they were embedded into paraffin blocks. The blocks were then sliced into 5–8 μm in thickness. The slices were in de-waxing with xylene and anhydrous ethanol and then stained with hematoxilin and eosin or with toluidine blue separately. For immunohistochemistry study, the dehydrated sections were incubated with primary antibody against type II antigens (diluted 1:200) overnight, then washed by TBST solution (pH 7.0) five times with 5 min per time, and incubated with antibody against collagen II (0.2%, Wuhan Boster Biotechnol. Co., Wuhan, China) for 30 min.

### Quantitative real-time PCR

Total RNA was isolated using Trizol (Invitrogen, USA) per manufacturer instructions. cDNA synthesis was done with random hexamerprimers. The gene expression was measured by quantitative real-time PCR (RT-PCR, Bio-RAD, USA) using SYBR method. For the in vitro experiment, the chondrocytes were digested in CO_2_ incubator under 37 °C for 4 min, stopped digestion by adding serum containing medicine, and counted the number as 9E5. The 3.3 ml of chondrocyte supernatant mixing with 14.7 ml medium was distributed into three 6-well plates, and every well contained 1.8E5 chondrocytes. After culturing for 3 days, the chondrocytes were separated with medium, mixed with 600 μl of RNAiso Plus, and transferred to centrifuge tube ready for PCR test. For the in vivo result, the frozen knee joint tissue was firstly treated by Trizol for 5 min, the supernatant was acquired by two times of centrifuge at 12,000 rpm for 15 min under 4 °C with chloroform emulsification in the middle, the bottom layer was separated by centrifuge of mixing original supernatant with isopropanol followed with washing by ethanol, and the liquid was sterilized and ready for next test. The 0.2 mL RNase free was mixed with the liquid on ice and treated under 37 °C for 15 min, with 85 °C for 5 s. Relative mRNA levels of target genes were normalized to GAPDH levels and further compared with the control using the 2-ddCt method. Designed primers were listed in Table 1.

**Table 1:** Primer sequence of RT-PCR

Primer name	Base sequence 5-3	Gene order
Collagen II	F:GAGGGCAACAGCAGGTTCAC	NM_012929.1
	R:GCCCTATGTCCACACCAAATTC	
Collagen II	F:CATGCCGTGACCTCAAGATG	NM_053304.1
	R:TCCATCGGTCATGCTCTCTC	
Aggrecan	F:TGGCATTGAGGACAGCGAAG	NM_022190.1
	R:TCCAGTGTGTAGCGTGTGGAAATAG	
β-actin	F:TTCAACACCCCAGCCATGTA	NM_031144.3
	R:CAGGAAGGAAGGCTGGAAGA	
SOX-9	F:GAAAGACCACCCCGATTACAAG	XM_003750950.3

### Primary chondrocytes culture

The primary chondrocytes were obtained from the articular cartilage of three newborn SD rats. All limbs were cut off from sacrificed newborn rats, and the shoulder, knee, and hip joints were immersed into Hank’s liquid and put into trypsin (GIBCO, USA) for digestion under 37 °C for 1.5 h, then centrifuged. The supernatant was discharged, and the sediment cells were mixed with 0.2% type II collagenase for 2-h incubation. The final sediment cells were washed two times and transferred into cell culture flasks to culture with Wistar rat serum containing APLD, GS, saline, or none, respectively.

The Wistar rat serum containing APLD, GS, saline, or none were firstly diluted to 10 and 20% separately. The diluted serum was used to incubate the SD rat chondrocytes in 96-well culturing plates under 37 °C. Each well was seeded around 6000 chondrocytes to grow. Then, chondrocytes were picked up and mixed with CCK-8 test kit (Dojin DO, China). The relating optical densities (OD) were measured each day for 4 days. The types and the concentration of the drug and time point were considered as three variances. Based on the OD values and corresponding variances, we figured out the optimized medicine concentration and the time point.

### Data presentation and statistical analysis

Statistical analyses were performed with the SPSS21.0. All data were presented as mean ± SEM. If the data from multiple groups match the independence, normal distribution, and homogeneity of variance, the data were analyzed with ANOVA followed with post hoc of LSD test to compare any two groups. If the data did not match the normal distribution and homogeneity of variance, Kruskal-Wallis test was used with Nemenyi test to compare each two groups. *P* values ≤ 0.05 were considered statistically significant.

## Results

### Radiographic imaging and analysis

The X-ray images showed the right knee joints at the time point of week 6 (immediately after surgery), week 10, and week 14 (that was after surgery 4 and 8 weeks).

At week 6, in group A, the joint gaps were visible, no bone hyperplasia could be seen, and homogeneous bone structure on the tibia proximal and distal femoral was clearly seen. No subchondral sclerosis and bone cyst were found, indicating no knee osteoarthritis in group A (Fig. 1a). In groups B, C, and D, we could see the joint gaps became narrow, the plane of tibia platform appeared coarse, and the femoral distal appeared sclerosis, indicating the early pathological characteristics [15, 16] of knee osteoarthritis (Fig. 1b–d).

At week 10, rats in group A showed a slightly narrow joint gap, the patellar and femoral surfaces were clear, no obvious hyperplasia around bones of joint, and no early symptoms of osteoarthritis (Fig. 1e). Knee joint gap was not entirely visible, and patellar/femoral joint surface basically disappeared in group B rats, indicating a typical mid stage of osteoarthritis (Fig. 1f). In groups C and D, knee joint gaps were narrow but did not disappeared, patellar outline was clear, and the patellar/femoral joint gaps were narrowing (Fig. 1g, h).

At week 14, in group A, knee joint gap did not show obvious changes, but visible tibia platform showed mild coarse and few sclerosis spots (Fig. 1i). In group B, the knee joint gaps were filled with bone spurs and sclerosis tissues, patellar/femoral contour was not clear, patellar/femoral joint gap disappeared, and the joint was obviously deformed, indicating a late stage of knee osteoarthritis (Fig. 1j). In group C, the knee joint gap was narrow, but did not entirely disappeared; the tibia platform showed sclerosis area, but the area was smaller than that in group B; and patellar profile was clear, but the patellar/femoral joint space narrowing, indicating a middle stage of knee osteoarthritis (Fig. 1k). The X-ray characteristics at this time in group D were similar with that in group C, but patellar/femoral joint space entirely disappeared (Fig. 1l).

### Histological results

**Fig. 2:**
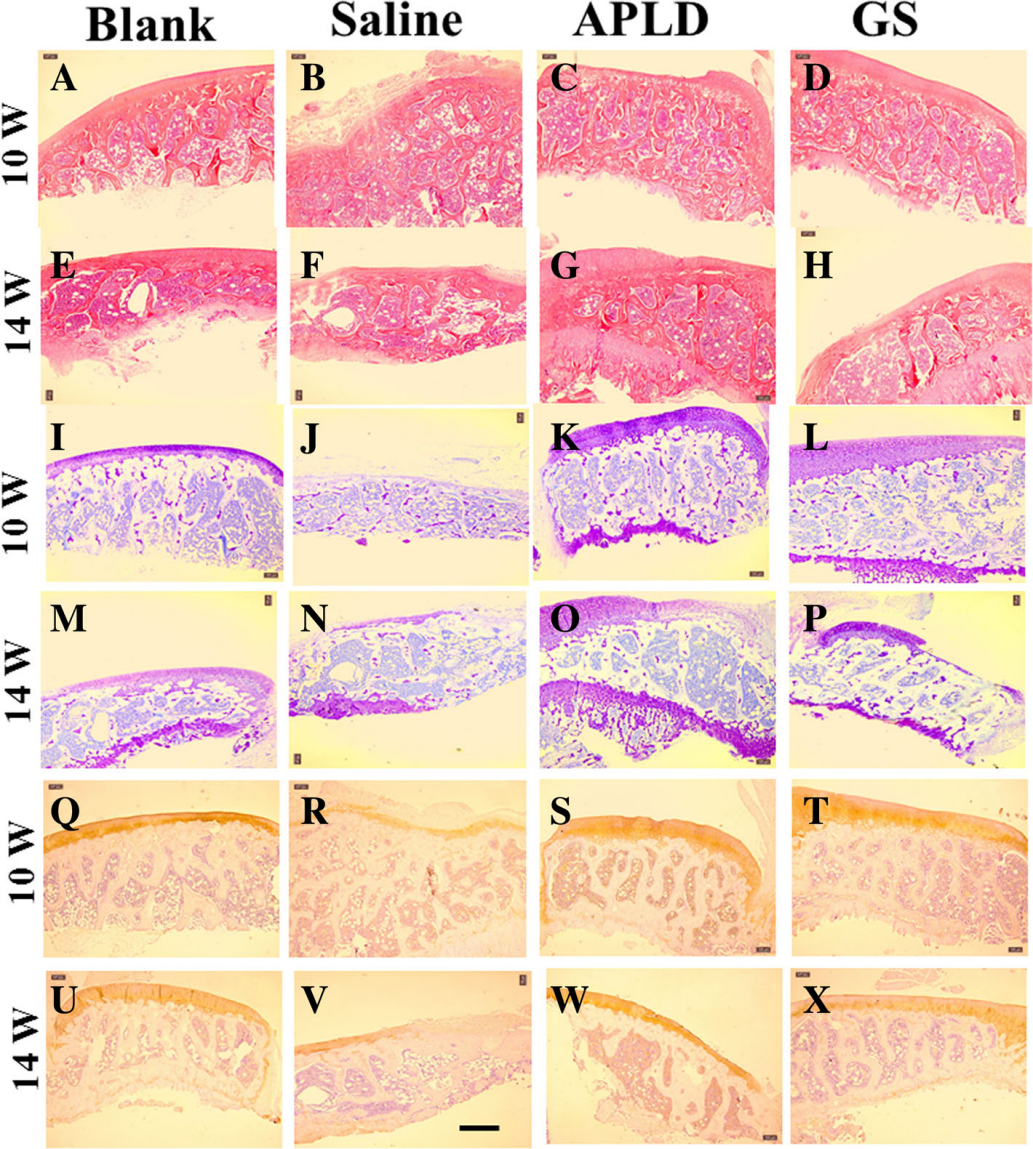
Right knee articular cartilage of histological imaging with staining of H&E, toluidine blue, and collagen II immunohistochemistry at weeks 10 and 14, which meant after medicine gavage treatment (4 and 8 weeks, respectively). **A**–**H** with H&E staining; **I**-**P** with toluidine blue staining; and **Q**–**X** with collagen II immunohistochemistry staining. Scale bar was 100 μm. Blank: group A; saline: group B; APLD: group C; and GS: group D

At week 10, knee articular histological structure in group A rats appeared clear and intact. However, knee articular histological structure in group B rats showed swelling and blurring; the surface of articular cartilage decayed and disappeared. To compare with group A, a slight swelling could be seen in the knee articular histological structure in groups C and D (Fig. 2a–d).

At week 10, we could see the significant atrophy and vacuolization in group B, congestion in group C, and no change in group D, when compared with imaging at week.

Toluidine blue staining is a semi-quantitative analysis of articular cartilage proteoglycan.

At 10th and 14th weeks, we could see that the proteoglycan levels and the surface of articular cartilage in group B were the least of all groups (Fig. 2j, n), while proteoglycan levels and the surface of articular cartilage in group C were the most of all groups (Fig. 2k, o).

Collagen II immunohistochemistry (shown as claybank color) could reflect the collagen II location and semi-quantitation of tissues. At 10th and 14th weeks, we could see the claybank color was apparently decreased in group B (Fig. 2r, v).

### Expressions of aggrecan and collagen II gene

Real-time PCR is a kind of DNA amplification reaction via fluorescent chemical signal to measure the total product after each PCR cycle. During the index of PCR amplification, Ct value of the template and the start copy number of the template exist the linear relation, which becomes the basis of quantitative analysis. The data of COL-II and ACAN expressions at No.4 week and No.8 week were shown as Tables 2 and 3 separately.

**Table 2:** Expressions of Collagen II and aggrecan genes from groups at 10th week (*n* = 3)

Gene	Group	$\overline{\mathrm{x}}$ ± s
Collagen II	A	1.04 ± 0.27
Collagen II	B	5.11 ± 0.38^a^
Collagen II	C	7.51 ± 1.73^a,b^
Collagen II	D	10.86 ± 0.07^a,b,c^
Aggrecan	A	1.00 ± 0.02
Aggrecan	B	3.36 ± 0.11^a^
Aggrecan	C	3.93 ± 0.27^a,b^
Aggrecan	D	5.51 ± 0.39^a,b,c^

All *P* < 0.01

^a^Compared with group A

^b^Compared with group B

^c^Compared with group C

**Table 3:** Expressions of Collagen II and aggrecan genes from the four groups at 14th week (*n* = 3)

Gene	Group	$\overline{\mathrm{x}}$± s
Collagen II	A	0.59 ± 0.23
Collagen II	B	0.39 ± 0.01
Collagen II	C	2.41 ± 0.50^a,b^
Collagen II	D	3.26 ± 1.75^a,b^
Aggrecan	A	0.62 ± 0.26
Aggrecan	B	0.15 ± 0.08
Aggrecan	C	2.08 ± 0.32^a,b^
Aggrecan	D	5.80 ± 0.61^a,b,c^

All *P* < 0.05

^a^Compared with group A

^b^Compared with group B

^c^Compared with group C

### CCK-8 test of chondrocytes

After comparing the OD values with group B (saline), the statistical results of groups C and D were shown in Table 4. Under the 20% concentration, the viabilities of both groups C and D were more than group B except day 2. The viability reached peak on day 3 and slightly decreased on day 4. Similar trend showed up under the 10% concentration; however, the viability of both groups were lower than group B on days 1 and 2. The result indicated that lower concentration of medicine could promote the proliferation of chondrocytes, and APLD could keep the viability of chondrocytes slightly better than GS since the cell number decreasing on day 4 was due to the inhibition of the cells following with full occupancy. We could conclude that the optimized interference time was day 3 and the concentration was 10%.

**Table 4:** CCK8 results of Statistical analyses (*n* = 3, $\overline{\mathrm{x}}$± s)

	20%		10%	
	Group C	Group D	Group C	Group D
1 day	1.29 ± 0.42	1.16 ± 0.61	0.77 ± 0.15^a^	0.62 ± 0.14^a^
2 days	1.28 ± 0.14^a, b^	0.85 ± 0.29^b^	0.92 ± 0.19	0.96 ± 0.25
3 days	1.39 ± 0.08^a, b^	1.15 ± 0.27^b^	1.28 ± 0.12^a^	1.28 ± 0.06^a^
4 days	1.06 ± 0.06^a^	1.08 ± 0.04^a^	1.15 ± 0.02^a^	1.18 ± 0.02^a^

Note: The value of Group B (SIA model control + saline) was considered as “1.” When the value of group C or D was more than “1,” it meant proliferation; when less than “1,” it meant suppression. It was clear that CCK8 value of the 10% was increased with the increased days in both groups, but no statistical significance between two groups

All *P* < 0.05

^a^Compared with group B

^b^Compared with group C

## Discussion

The study results showed the levels of collagen II and aggrecan were significantly higher in group C or D than in group A, and were significantly higher in group C than in group D. The higher level of collagen II expression in groups C and D may indicate the function of SOX9 in the process of cartilage chondrocytes maturation and differentiation because of the consistent work of collagen II with SOX9. In addition, the level of aggrecan expression was higher in group C than in group D, which supported the multiple target genes of APLD. APLD had the advantage on multiple target gene [16]; however, further studies would be required to figure out in which target gene and signal path the APLD works on.

Knee joint cartilage is a kind of hyaline cartilage and located on the surface of the femur bone. Because the knee joint cartilage tissue lacks the nerve, vessel, and lymph-vessel, it can only receive nutrition components from synovial fluid [17]. This characteristic limits the self-healing of knee joint cartilage after lesion. If the pain apparently appears in the knee of a patient, it indicates that the KOA has developed into middle or late stage and the knee joint cartilage tissue may not be able to reach the self-healing. The collagen II begins to denature, dissociate, and decrease, which leads to the corrupt, destroyed, and even disappearance of fibrous structure. Meanwhile, the aggrecan fraction in the knee joint tissue shrinks down, leading to the ECMC shrink down and finally to the decreased and degenerated cartilage chondrocytes. That is part of the reason of irreversible translation from compensatory to de-compensatory effect. From our study of rat KOA models, we found that APLD and GS treatments were able to prevent the shrinking down and loss of collagen II and aggrecan, to slow down the development of KOA, and to keep the knee joint tissue within the control of compensatory effect. We did not observe that APLD treatment showed any adverse effect to the viability of cartilage chondrocytes. In addition, we noticed that tissue structure was apparently loosened histologically in each group with different staining at week 14 compared with week 10, which may indicate the aging process (Fig. 2). The efficacies of medicine treatment were clearly observed on X-ray images at week 14, in which both knee joint gap and bone structure (such as the compact bone and the cancellous bone in the tibia) disappeared in group B rats (Fig. 1j), but they were visible in group A, C, and D rats (Fig. 1i–l). Histological images with three types of staining revealed that the bone tissue structure and the composition of knee joint showed the clearness without degradation and shrinking in group C (Fig. 2c, g, k, o, s, and w).

## Conclusion

Based on the result above, we concluded that APLD or GS gavage treatment for KOA rat models was effective in terms of the proliferation of cartilage chondrocytes and the damaged knee joint tissue repairing, and the APLD showed slightly superior in general.

## Abbreviations

APLD: Pubescentis and Loranthi decoction
GS: Glucosamine sulfate
IACUC: Institutional Animal Care and Use Committee
KOA: Knee osteoarthritis
OD: Optical densities
SIA: Surgery-induced arthritis
TCM: Traditional Chinese medicine
TE: Tissue engineering
